# An Empirical Study on the Impact of Different Interaction Methods on User Emotional Experience in Cultural Digital Design

**DOI:** 10.3390/s25175273

**Published:** 2025-08-25

**Authors:** Jing Zhao, Yiming Ma, Xinran Zhang, Hui Lin, Yi Lu, Ruiyan Wu, Ziying Zhang, Feng Zou

**Affiliations:** 1 College of Art and Design, Beijing University of Technology, Beijing 100124, China; zhaoj@bjut.edu.cn (J.Z.); mym1202@emails.bjut.edu.cn (Y.M.); zhangxinran02@emails.bjut.edu.cn (X.Z.); linhhui6828@emails.bjut.edu.cn (H.L.); ziyingzhang@emails.bjut.edu.cn (Z.Z.); 2 College of Applied Arts and Science, Beijing Union University, Beijing 100101, China; 20232035110104@buu.edu.cn

**Keywords:** cultural heritage, digital interaction design, user emotional experience, functional near-infrared spectroscopy, infrared thermography, electrodermal activity

## Abstract

Traditional culture plays a vital role in shaping national identity and emotional belonging, making it imperative to explore innovative strategies for its digital preservation and engagement. This study investigates how interaction design in cultural digital games influences users’ emotional experiences and cultural understanding. Centering on the Chinese intangible cultural heritage puppet manipulation, we developed an interactive cultural game with three modes: gesture-based interaction via Leap Motion, keyboard control, and passive video viewing. A multimodal evaluation framework was employed, integrating subjective questionnaires with physiological indicators, including Functional Near-Infrared Spectroscopy (fNIRS), infrared thermography (IRT), and electrodermal activity (EDA), to assess users’ emotional responses, immersion, and perception of cultural content. Results demonstrated that gesture-based interaction, which aligns closely with the embodied cultural behavior of puppet manipulation, significantly enhanced users’ emotional engagement and cultural comprehension compared to the other two modes. Moreover, fNIRS data revealed broader activation in brain regions associated with emotion regulation and cognitive control during gesture interaction. These findings underscore the importance of culturally congruent interaction design in enhancing user experience and emotional resonance in digital cultural applications. This study provides empirical evidence supporting the integration of cultural context into interaction strategies, offering valuable insights for the development of emotionally immersive systems for intangible cultural heritage preservation.

## 1. Introduction

Traditional culture serves as a crucial vehicle for national identity, historical memory, and spiritual values, playing an irreplaceable role in cultural continuity. However, due to a lack of immersive experiences and weakened dissemination, traditional culture today often suffers from insufficient public cultural identity, leading to a disconnection in cultural inheritance. In particular, younger generations such as university students, despite being digitally native, often exhibit a disconnect from traditional cultural expressions. Thus, revitalizing traditional culture in a way that resonates with young people has become a pressing issue for contemporary cultural design.

In recent years, the development of digital interaction technologies has enabled traditional culture—especially intangible cultural heritage (ICH)—to be embedded into interactive systems in engaging and participatory ways, thereby integrating into everyday life [1,2]. These practices collectively demonstrate that digital interaction has become an important avenue for cultural innovation. Beyond enabling performance, interaction, perception, experience, response, and reflection, such technologies profoundly influence users’ understanding, internalization, and emotional identification with cultural information.

Currently, digital interaction design for cultural heritage is transitioning from “content presentation” to “experience construction”. The prevailing approaches mainly involve the digital preservation and presentation of cultural artifacts, such as cultural databases and digital archives, interactive exhibitions, multimedia guides, and immersive experiences, using virtual reality (VR), augmented reality (AR), and other interactive technologies [3]. Designs aimed at cultural preservation focus on the visual representation and storage of cultural data, while those targeting cultural education emphasize user participation by integrating cultural elements into interactive scenarios or simulating cultural behaviors to enhance cultural perception and identification [4].

However, the current digital interaction design of cultural heritage still faces limitations in interaction modalities. Most culture-based digital systems rely on generic interaction paradigms. They do not adequately consider cultural–contextual fit, which refers to the organic integration between interaction technology and cultural characteristics. As a result, the digital expression of culture often remains at the level of symbolic representation or functional behavior replication. Interaction modalities not only affect usability but also deeply shape users’ cultural cognition and emotional experiences [5]. Emotional experience involves the users’ emotional engagement, sense of immersion triggered by cultural stimuli, and physiological arousal [6,7,8]. The cultural appropriateness, embodied fidelity, and immersive potential of an interaction design directly influence the acceptance and internalization of cultural meaning. From the perspective of embodied cognition, interaction modalities that align with culturally familiar gestures or bodily movement not only support intuitive usability but also activate perceptual–motor systems that ground cultural symbols in bodily experience, thereby enhancing emotional experience [9]. In addition, the field of affective computing emphasizes that sensor-based platforms (e.g., facial EMG and gesture kinematics) can provide real-time indicators of emotional engagement in cultural interactions [10]. Thus, by bridging physical interaction, bodily simulation, and emotional arousal, culturally congruent interaction can more deeply shape cultural cognition and emotional experience.

Traditional user experience evaluation methods have mostly relied on subjective approaches like questionnaires and interviews. There has been limited use of physiological data for multimodal assessment. This makes it difficult to capture users’ genuine emotional fluctuations and cognitive load [11,12]. Recent studies in player experience and game user research have begun to adopt physiological measurements, such as fNIRS, eye tracking, and EDA, in conjunction with subjective assessments to create more comprehensive evaluation systems [13,14]. In particular, research in immersive environments has shown that combining fNIRS with IRT and EDA can effectively track users’ real-time stress levels, engagement, and cognitive load [15]. These approaches provide methodological inspiration for building multimodal and developmentally sensitive evaluation systems in interactive cultural experiences. Nevertheless, there remains a lack of systematic research employing multimodal methods that focus specifically on cultural interaction experiences. Therefore, how to design more immersive interactive methods in the digitalization of culture and evaluate user experience with more scientific and comprehensive approaches requires in-depth exploration.

In light of these challenges, our research addresses the following core question: In cultural theme interaction designs, what types of interaction modalities are most effective in eliciting emotional responses and enhancing cultural experiences? We select the traditional puppet art of marionette puppetry, a form of intangible cultural heritage, as the thematic foundation for developing a cultural experience game. We then evaluate university students’ emotional experiences across three interaction modes: Leap Motion-based gesture interaction, keyboard interaction, and non-interactive viewing.

Using a within-subjects experimental design, we integrated multiple measurement methods, including fNIRS, infrared thermography, EDA, and subjective questionnaires, to analyze users’ emotional responses, immersion levels, and cultural perceptions under different interaction conditions. fNIRS was selected as a core tool for assessing emotional and cognitive responses due to its lower sensitivity to motion artifacts and its suitability for detecting brain function in naturalistic settings—advantages over positron emission tomography (PET), electroencephalography (EEG), and functional magnetic resonance imaging (fMRI) [16]. Through this research, we aim to construct a causal link between the cultural congruence of interaction modalities and users’ emotional experiences, thereby providing empirical evidence and design guidance for future cultural interaction design and cultural communication practices.

In the remainder of this paper, Section 2 reviews two relevant research domains: (1) interaction design that accounts for cultural characteristics and (2) evaluation methods using multimodal physiological data. Section 3 outlines our experimental setup, including materials, participants, measurement techniques, and procedures. Section 4 presents the results, followed by a discussion in Section 5 and conclusions in Section 6.

## 2. Related Work

### 2.1. Research on Digital Interaction Design for Cultural Heritage with Cultural Characteristics as a Key Consideration

In recent years, digital interaction design related to cultural heritage has gradually become an important issue in the fields of intelligent interaction and cultural heritage revitalization. However, while existing research generally acknowledges the role of cultural elements in user experience, there are still limitations in the design of interaction modes.

On one hand, most studies primarily integrate cultural elements into visual styles, symbolic representations, and contextual construction, neglecting the cultural expression potential inherent in the interaction modes themselves. On the other hand, emerging technologies such as VR and artificial intelligence (AI) are increasingly applied in the digital expression of cultural heritage. However, most of these studies primarily emphasize the immersive experience and technical innovation of digital presentations. In contrast, relatively few explore how users perceive cultural meaning or how emotional resonance is triggered during interaction.

A large number of existing studies mainly focus on the reproduction of cultural content in practice, particularly in terms of visual and contextual design. For example, Muntean et al. [17] embed the cultural values of the Masqun ethnic group into an interactive physical table as part of an exhibition setup, attempting to guide users in perceiving cultural narratives, but the interaction logic still relies on traditional click-and-browse operations. Sun et al. [18], in their development of a VR system based on Dunhuang culture, stimulate user interest through storytelling and multimodal presentations. However, the core of the system is still a technology-driven experiential flow, rather than an interaction logic driven by culture. Li et al. [19] explore how visual images and human–computer interaction can be combined in cultural creative product design. They propose several ideas related to cultural visual interaction. However, their approach remains centered on functional operations and lacks a clear mapping to cultural behavioral logic.

Further, some studies try to reflect cultural features in interaction design. However, they often focus on sensory stimulation and ease of use, without integrating cultural thinking patterns or embodied behaviors linked to heritage practices. For example, Long et al. [20] introduce traditional Chinese cultural gestures on mobile devices, using tangible gestures to evoke users’ sense of identity and cultural emotions. However, they also point out that there is still a lack of systematic construction and mapping mechanisms for cultural cognitive models, making it difficult to form stable cultural behavioral inertia. In culturally related digital games, the most commonly used interaction modes are key presses or clicks, but for the types of cultural heritage that rely on fine motor skills or physical participation, these modes cannot reproduce the rhythm of movement and bodily gestures, leaving the user experience in a passive reading or viewing stage. In other words, the existing interaction modes are largely “representational inputs” of cultural symbols, rather than “behavioral embedding,” failing to reach the deeper psychological channels of cultural identity.

Moreover, with the development of mixed reality (MR), motion-sensing technologies, and other innovations, an increasing number of projects are attempting to build immersive cultural heritage experience systems. However, these often fall into a “technology-centered” tendency in interaction design. In such studies, interaction modes typically serve technological display rather than cultural expression. For example, the mixed reality puppet performance system developed by Lin et al. [21] and the interactive museum design at the Florence Cathedral by Rinaldi et al. [22] both demonstrate the richness of cultural experiences, but the system interaction designs often lack a deeper construction of the cultural experience mechanisms. This emphasis on display over construction in the interaction path can turn users into “cultural spectators” rather than “cultural participants,” making it difficult to establish sustained cultural emotional connections.

In conclusion, while current research has begun to recognize the importance of cultural factors in interactive experiences, most studies still focus on superficial cultural content transmission and esthetic interface creation. Research that truly uses cultural characteristics as a guiding force to explore the mapping and coupling between interaction modes, cultural behaviors, and cultural traits remains scarce. Interaction modes are not only media for information transmission but also key channels for users’ cultural perception, identity construction, and emotional resonance. Related research in the field of cross-cultural human–computer interaction emphasizes that interface gestures, timing, and affective cues must resonate with users’ cultural schemas to elicit meaningful emotional and identity-oriented responses [23]. Together, relevant research on embodied cognition [24] and affective computing shows that by understanding visitors’ personal cognitive needs and interests and their situational affective states [25], as well as choosing interactive methods for embodied cultural heritage experiences, it is possible to enhance visitors’ emotional experience and cultural identity and bridge the emotional gap [26]. These studies show that interaction modes do not merely execute tasks but can elicit culturally patterned cognitive–emotional responses through bodily simulation, mirror-resonance, and sensorial grounded meaning. Accordingly, how to reflect the logical structure of cultural behaviors, value systems, and emotional expressions in interactive mechanisms remains an important area in the digital interaction design of cultural heritage that requires deeper exploration.

### 2.2. Research on User Emotional Experience Evaluation Driven by Multimodal Physiological and Psychological Data

As the role of emotional experience in human–computer interaction (HCI), immersive systems, and digital product design becomes increasingly prominent, more and more studies are attempting to explore how to assess users’ emotional states in a more comprehensive and objective manner. Especially in complex interactive environments such as immersive experiences, VR, and gaming, single subjective assessments often fail to fully reflect users’ true emotional states. The introduction of physiological signals has become a key approach to enhancing the accuracy and objectivity of assessments. However, despite the practical advancements in physiological measurements, most current research still limits its use to supplementary methods. The true integration of subjective psychological indicators with objective physiological data, and the construction of a systematic, multimodal emotional assessment framework, remains a core challenge in current research.

In existing evaluation methods, subjective tools still dominate. A range of tools—including traditional emotional self-report questionnaires (e.g., REQ) [27], task-specific experience measures, and design-oriented methods like Mood Boards [28]—highlight the user perspective and regard subjective feelings as a primary basis for understanding emotional experience. These methods are low-cost, easy to implement, and especially suitable for early prototype testing and effective design validation. However, these approaches are limited by factors such as users’ self-expression abilities and subjective awareness, making it challenging to capture nuanced and dynamic emotional changes. Furthermore, users’ subjective expressions are often influenced by cognitive biases, language abilities, and social expectations, making it difficult to provide continuous and objective data support.

In response to these methodological limitations, researchers have begun to introduce physiological signals as an important supplement to emotional assessment, such as heart rate, EDA, facial expressions, and EEG, to enhance the objectivity of evaluations. The core value of these methods lies in their ability to capture immediate, subconscious emotional responses. For example, Liapis et al. [29] developed the PhysiOBS system, which combines physiological sensors with behavioral observation and self-reporting to achieve multidimensional assessments of users’ emotional states, demonstrating the potential of multimodal collaborative analysis. Similarly, Barrow [27] attempted to combine physiological arousal indicators with self-assessment in predicting emotional responses. The emergence of such tools indicates a shift in user experience evaluation from static measurement to dynamic perception. However, current physiological measurement methods still mainly focus on single-modal data collection, with limited integration of multimodal physiological data analysis.

Beyond traditional affective computing tools, sensor-based approaches have further extended emotional evaluation frameworks in interactive and developmentally sensitive environments. For example, real-time changes in skin conductance have been used to assess emotional arousal, decision-making pressure, and cognitive effort during gameplay and interactive learning [21,22,23,24,25,26,27,28,29,30,31,32,33]. Facial thermography and muscle activity (EMG) also offer insights into subtle emotional shifts under stress or surprise [34]. These physiological markers, when combined with subjective reporting, provide a more nuanced depiction of emotional intensity, engagement density, and behavioral readiness, especially in culturally rich or sensorimotor-driven contexts. The advancement of these sensor-based measurement techniques supports a more reliable and multidimensional understanding of user states in culturally immersive experiences.

In HCI and HMI research, as interaction methods become more diverse and complex, the demand for multimodal data fusion is also growing. However, existing practices related to physiological signals, such as heart rate and electrodermal activity, are mainly used to reflect immediate emotions, such as pleasure, not to effectively cover multidimensional psychological states, such as cognitive load and cultural contexts. For instance, Abriat A et al. [35] integrate behavioral tools (such as pleasure scales) with physiological signals (EMG, RR) to assess the skincare product usage experience in menopausal women, but they still focus on a single emotional dimension, such as “pleasure.” Liu et al. [36] use eye-tracking and emotional questionnaires to assess the public’s esthetic responses to streetlamp designs. Although they introduce multimodal tools, the analysis metrics remained limited to emotional responses at the surface level, without addressing the deeper cultural cognition and resonance mechanisms of users.

In summary, the integration of subjective and objective data in multimodal emotional experience assessments has become an important direction in interactive experience research. To further enhance the cultural adaptability and psychological recognition dimensions of these assessments, this study aims to systematically assess whether culturally adapted interactions can significantly enhance users’ emotional experience quality and cultural identity, based on subjective evaluation and multimodal physiological data (such as fNIRS HbO concentration, facial temperature, EDA, etc.).

## 3. Materials and Methods

### 3.1. Participants

To investigate users’ emotional experiences in a cultural game with different interaction modalities, the participants are recruited via the Wenjuanxing v2.2.6 program on WeChat v1.0.9 between April and August 2025. This study was approved by the Beijing University of Technology Ethics Committee. Written informed consent was obtained from all participants prior to data collection. Participants who successfully completed the experiment received a monetary reward of 20 RMB. A total of 66 volunteers participated in the study. Due to unexpected instrumental interruptions and accidental loss of certain channels in fNIRS recordings, complete and analyzable datasets were ultimately obtained from only 61 participants. We also administered a pre-experiment questionnaire to collect participants’ background information. In addition to gender, age, and academic major, the questionnaire includes items assessing participants’ familiarity with Chinese traditional culture and digital gaming experience to capture their cultural cognition and operational competence. Questionnaires are scored using a Likert scale (1 = not at all, 5 = very).

We conducted basic descriptive statistical analysis of the questionnaire results, see Table 1. The results show that all participants are full-time college students, ranging in age from 18 to 28 years old, with undergraduate, master’s, and doctoral degrees. Their majors include electronic engineering, computer science, industrial design, mechanical engineering, etc. This diversity is intended to ensure a wide range of perspectives and evaluations during the cultural game experience. We also calculated the mean (M) and standard deviation (SD) of key variables. Participants report an average familiarity with Chinese traditional culture of M = 2.18, SD = 0.74, indicating a moderate cultural background. Their average weekly gameplay time is M = 11.37 h, SD = 10.46 h. About 75% report prior experience with PC or console games, but only 18.03% have used Leap Motion or similar gesture-based devices. These results show that participants generally possess sufficient gaming skills to complete the tasks. Meanwhile, cultural familiarity reduces the risk of misunderstanding the content of game. Limited exposure to gesture interaction also minimizes familiarity bias in the Leap Motion condition. Therefore, participants’ background variables exert minimal influence on the internal validity of this study. As most participants have a comparable baseline in cultural understanding and operational ability, observed emotional and physiological differences are likely attributable to the interaction modality than to individual variation.

The experiment follows a single-blind design, in which participants are unaware of the research hypotheses and expected outcomes, thereby minimizing subjective bias. This participant setup provides a stable and representative data foundation for the subsequent multimodal data collection and comparison across interaction modalities.

### 3.2. Materials

In this study, the Quanzhou String Puppet is selected as the cultural carrier based on the digital interactive design of ICH. As an ancient form of traditional folk art, Quanzhou string puppetry is characterized by profound cultural attributes and distinctive artistic features [37]. The structure of a typical string puppet consists of the puppet head, torso, limbs, control rods, and strings. Each puppet is equipped with more than a dozen to several dozen strings, meticulously connected to various joint parts, forming a complex and precise string control system. This system enables flexible joint movement and allows for highly refined motion control through string manipulation [38]. The operational uniqueness of Quanzhou string puppetry lies in the intricate string system and the highly skilled string-handling techniques. During performance, puppeteers must precisely modulate the tension and rhythm of each string using delicate coordination of fingers and wrists, thereby enabling the puppet to exhibit a lifelike gait and expressive movements [39].

Unlike most static or visually dominant forms of ICH, string puppetry emphasizes dynamic manipulation and embodied performance, relying on fine motor skills and long-term embodied memory. The mapping between the puppeteer’s hand movements and the puppet’s actions is fundamental to its cultural expression mechanism. Given the unique operational logic embedded in this traditional heritage, its digital reinterpretation necessitates an interaction modality that maintains both cultural fidelity and operational authenticity. The goal is to construct a digital interaction system capable of fostering physical engagement and re-enacting traditional manipulation techniques, thereby unlocking the embodied cultural experience embedded in this art form.

Based on these cultural performance characteristics, a Leap Motion gesture recognition device is introduced into the experiment as a culturally consistent gesture interaction paradigm. This setup enables users to control the digital puppet through natural hand and wrist movements, simulating traditional string manipulation. Through micro-operations of fingers such as opening and closing, rotating, pushing, and pulling, key movements such as puppet walking, jumping, and waving can be achieved. To allow for comparative analysis, two additional interaction conditions are implemented: keyboard-based interaction and non-interactive viewing. This comparative framework enables us to explore the relationship between interaction modality and user experience.

All interaction prototypes are developed using the Unity3D engine (version 2023.3.0f1c1). The tasks and visual content are kept consistent across all interaction conditions to minimize confounding effects from content variation. The core mechanic requires users to control puppet actions to perform designated cultural movements, preserving the symbolic cultural logic of traditional puppetry while enabling controlled manipulation of interaction variables.

**Figure 1:**
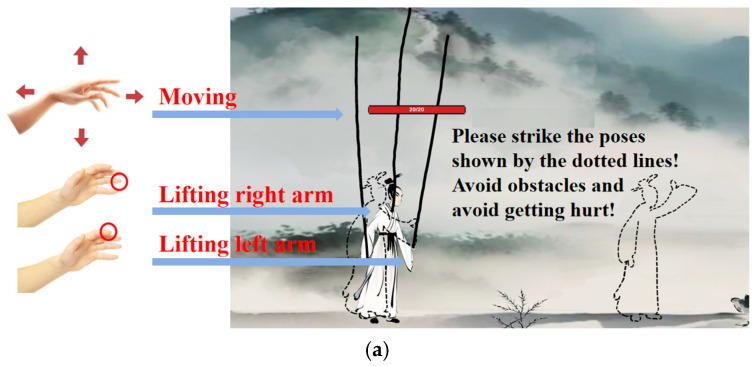

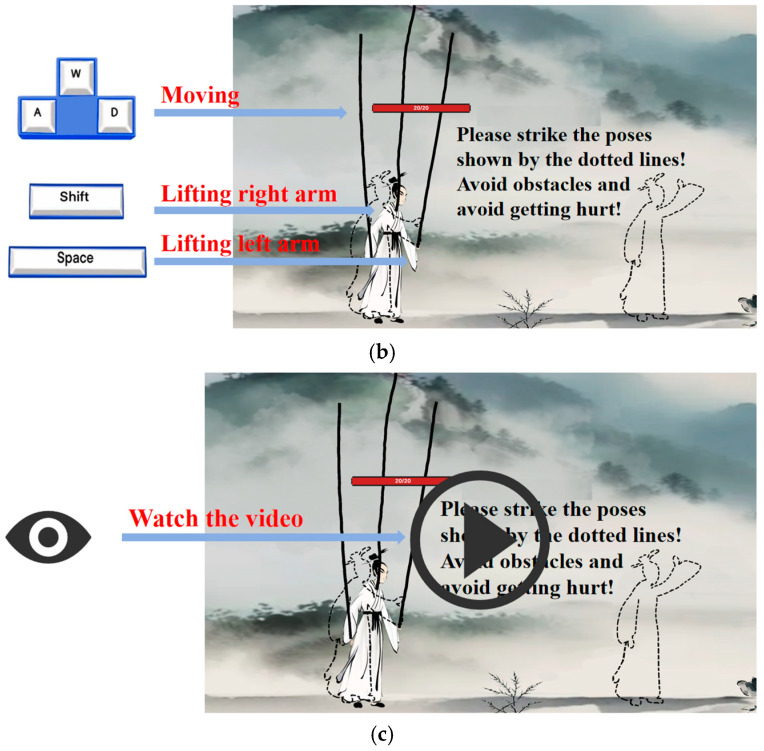
Schematic diagram of interaction mode: (**a**) gesture interaction; (**b**) keyboard interaction; (**c**) no interaction.

As illustrated in Figure 1, the three interaction modalities vary in terms of their logical and cultural congruence:

- Gesture Interaction: Gesture interaction allows players to engage with the game using intuitive, natural gestures that simulate real-world puppet manipulation. It offers high immersion and strong cultural alignment.
- Keyboard Interaction: Keyboard interaction employs traditional key inputs to control the puppet’s arm movements. While moderately immersive, it lacks the physicality and cultural resonance of traditional manipulation.
- No interaction: The non-interaction mode is to let participants simply watch a prerecorded gameplay video without any user input, representing a passive mode of cultural reception with low engagement and limited cultural transmission.

To enable smooth and natural gesture interaction, the Leap Motion controller is used for hand-tracking. This device employs high-precision infrared sensors to capture users’ hand movements in real time without noticeable latency. Specifically, the height of the user’s index and ring fingers is mapped to the puppet’s right and left arm movements, as shown in the red circle in Figure 1(a). The position of hand determines the puppet’s spatial coordinates within the game environment. Compared to conventional motion tracking solutions, Leap Motion offers advantages such as portability, high responsiveness, and intuitive operation, making it particularly suitable for interactive scenarios that require detailed hand motion, like string puppetry. In contrast, the keyboard-based control uses the Shift key for left arm movement, the Space bar for right arm movement, and the “A” and “D” keys for directional navigation. The non-interactive condition involves a timed playback of a prerecorded gameplay session.

To ensure that the gesture design and narrative expression of the digital puppet system avoid cultural misrepresentation, a validation process is conducted through expert review and literature-based justification.

Semi-structured interviews are conducted in our research for the reason that they are flexible and also applicable. An outline of the interview could be drawn up in advance, but it is not necessary to follow it completely, and it can be adjusted flexibly according to the interview subjects, the process, and content [40]. Therefore, this study adopts semi-structured interviews with highly representative stakeholders to obtain the most direct and honest perception of the cultural experts on the current status and shortcomings of the digital design of Puppetry. Three categories of stakeholders have been selected: The Puppetry expert performer, the Puppetry show organizer and manager, and also researcher focused on cultural heritage. The details of the respondents are shown in Table 2.

The interviews are conducted through the Tencent Meeting online platform. Each one takes around 30 min. Firstly, we inquire about the background information. Then, after showing the digital game developed by our team, as well as the one used as the study material in our experiment, questions about the Puppetry gestures, overall narrative, and digital form are put forward to gain their insights on whether the gesture–action mappings and the narrative structure of the digital puppetry system align with the traditional string puppet. The specific questions are shown in Table 3.

All three experts gave positive feedback on these questions. They confirm that the design retained the expressive qualities of traditional string puppetry and represented a culturally recognizable form of performance such as the gestures and ways of operating. Although the game is presented in a relatively simple way, it offers novel insights and a creative route for developing traditional Puppetry, especially regarding the needs of the audience of the young generation. In actual performances, the number of strings is usually large and the manipulation methods are also more complex. However, this digital game is sufficient for testing the form and is a valuable way to promote the communication of traditional Puppetry. Their attitude reflects a high degree of cultural consistency and endorsement of our digital approach. They also expressed approval of the narrative of the game. They are embracing and welcoming new repertoire stories that are rooted in our society. All three experts highly praise the new digital approach to redesigning Puppetry culture. They also provide further suggestions, such as diversifying the types of puppet shows by incorporating Glove Puppet shows and Rod Puppet shows, as string puppetry is typically popular in the southern regions of China, while rod puppetry is more commonly disseminated in the northern part. Designing distinct digital interaction methods based on different types of puppets will enhance cultural dissemination effectiveness and broaden audience reach.

Additionally, the design approach is supported by prior research on digital puppetry systems. Previous studies confirm that well-designed interactive and gesture-based systems are capable of preserving essential cultural elements [41]. For instance, Zhang demonstrates that hand gestures such as opening and clenching can replicate traditional manipulation techniques like twisting and rubbing, thereby maintaining performative authenticity [42]. Antonijoan et al. show that tangible puppets controlling virtual avatars help expand narratives without distorting cultural meaning [43]. Wang further concludes that digital puppetry systems support embodied learning and contribute to the transmission of traditional skills and knowledge [44]. These findings reinforce the cultural validity of the proposed system design.

To ensure that the gesture-based interaction does not introduce discomfort or excessive task demands that could confound the interpretation of physiological responses (e.g., mistaking discomfort for emotional arousal), a usability evaluation is conducted. The evaluation framework is informed by gestural interaction usability heuristics [45] and adapts quantitative measures from established gesture interface evaluation studies [46,47]. Three critical dimensions are examined: learnability, cognitive load, and fatigue. See Table 4 for the usability evaluation scale. A total of fourteen 5-point Likert scale items are used, with scores ranging from 1 (“strongly disagree”) to 5 (“strongly agree”). Positive items are reverse-coded to ensure that lower scores consistently indicate better usability. The median reference value is set to 3 for subsequent non-parametric testing.

Internal consistency of the scales is satisfactory to excellent, with Cronbach’s α of 0.746 for learnability, 0.750 for cognitive load, and 0.814 for fatigue (overall α = 0.832), as shown in Table 4. Normality tests (Shapiro–Wilk) indicate that all item distributions significantly deviate from normality (*p* ≤ 0.05); thus, Wilcoxon signed-rank tests with continuity correction (10,000 iterations) are applied to compare each item’s distribution against the median. Table 5 summarizes descriptive statistics and test results for each item.

Results indicate that all learnability items scored significantly below the median (all *p* < 0.05 and all Z < 0, the Z-score is a standardized test statistic used to measure the direction and strength of systematic deviations). For the reason that learnability is verse coding (1 for most learnable and 5 for not learnable at all) to stay consistent with the other two dimensions, it suggests that participants found the gesture interaction intuitive and easy to understand from the outset. Within the cognitive load dimension, most subscales are significantly below the median except mental demand (C1) and time demand (C3) (*p* < 0.05 and Z < 0), indicating that the gesture interface imposed a low cognitive load during use. All fatigue-related items are significantly below the median (all *p* < 0.05 and all Z < 0), with particularly low scores in energy depletion (F4) and motivation drop (F5), suggesting that the gesture interface does not cause notable physical or mental fatigue even during sustained use.

Taken together, the findings demonstrate that the gesture interface achieves high learnability, imposes minimal physical and mental strain, and maintains ergonomic comfort. This supports the conclusion that participants’ physiological responses in the main experiment are unlikely to be confounded by discomfort or usability issues, reinforcing the ecological validity of the emotional engagement results.

Overall, the design aims to simulate real-world cultural practice processes while controlling for experimental consistency. This approach maximizes the comparability of emotional, cognitive, and physiological responses elicited by different interaction modalities. The findings serve as an empirical foundation for future studies on the role of cultural congruence and interactional immersion in shaping user emotional experiences in culturally themed digital interaction design.

### 3.3. Experimental Procedure

**Figure 2:**
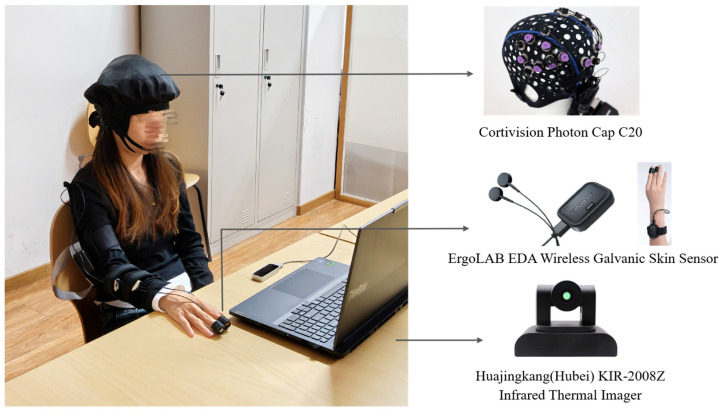
Experimental scene and equipment used.

This study adopts a within-subject experimental design, in which each participant is asked to evaluate their personal gameplay experience with the Leap Motion system, keyboard interaction, and non-interactive viewing in order to determine individual preferences. To counterbalance potential carryover effects inherent in repeated-measures designs, participants are randomly assigned to different groups and exposed to the three systems in varied sequences, as illustrated in Figure 2. Specifically, a randomized sequence list of the three interaction modes was generated using an online random sequence generator prior to the experiment, and each participant was assigned a corresponding experience order accordingly. This approach effectively mitigates potential fatigue or learning effects associated with fixed sequences in within-subject designs, thereby improving the validity of the data and ensuring the fairness of cross-condition comparisons.

**Figure 3:**
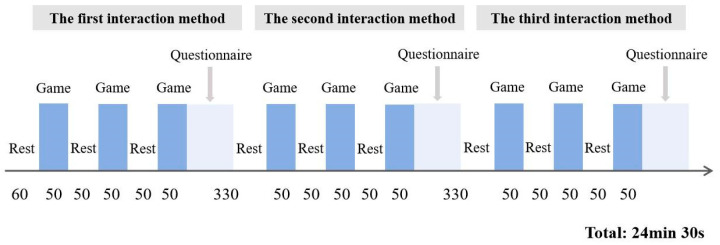
Experimental procedure.

Prior to the experiment, administrators assist participants with the setup and calibration of physiological monitoring equipment, including an fNIRS device, EDA sensors, and an infrared thermal imaging camera. The entire experimental procedure is conducted under the supervision of two administrators and consists of the following six steps, as shown in Figure 3.

Step 1: Participants are asked to provide informed consent for the use of fNIRS, EDA sensors, and an infrared imaging camera to record their physiological responses throughout the experiment.

Step 2: Administrators introduce the task for each cultural experience game: “Use your hands or the keyboard to control the character’s movements. When the character reaches the designated position, the level is completed.” For the non-interactive condition, “Simply watch the video. No action is required.”

Step 3: Participants are engaged with the first assigned cultural heritage interaction game for 50 s, followed by a 50 s rest period. This process is repeated three times. During this phase, the fNIRS and EDA sensors continuously and automatically record participants’ physiological activity, while the infrared imaging is manually operated and recorded by the experimenter using specialized software.

Step 4: After completing the first task, participants rest for 5 min before proceeding to the next interaction condition, repeating the procedures outlined in Step 3.

Step 5: After each interaction session, participants are given a 5 min interval to complete a subjective gameplay experience questionnaire, assessing their perceived experience during the game.

Step 6: Upon completing all three interaction conditions, participants are asked to indicate which cultural heritage experience game they prefer and explain why. The rationale for asking a comparative preference question rather than an absolute evaluation is to elicit more authentic insights into user preferences through relative judgments, which tend to produce more reliable results.

**Figure 4:**
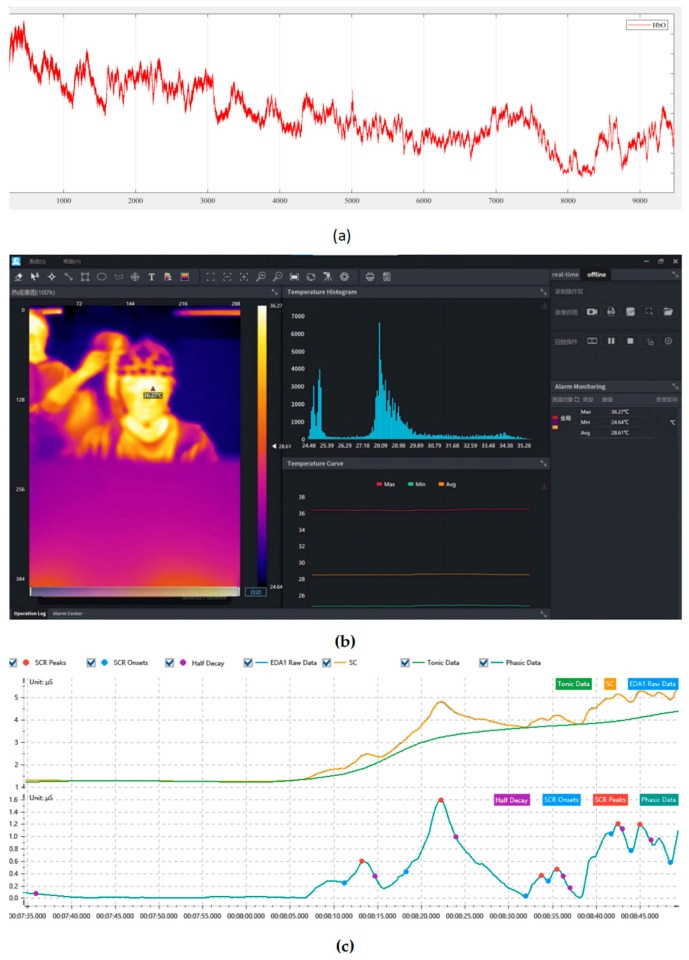
Schematic representations and sample waveforms of physiological signal types: (**a**) changes in cerebral oxygenated hemoglobin concentration; (**b**) temporal variation in skin temperature; (**c**) variations in heart rate and electrodermal activity signals.

Throughout the experiment, two categories of data are collected: physiological signal data and subjective rating data. The physiological signals comprise three modalities: fluctuations in cerebral oxygenation obtained through fNIRS, variations in skin conductance recorded by EDA sensors, and thermal distribution images of the facial regions captured using an infrared thermal imaging camera. All physiological data are recorded as continuous time-series signals. The subjective data consist of questionnaire scale scores. Figure 4 presents schematic representations and sample waveforms corresponding to the three physiological signal types.

The synchronized acquisition and analysis of both physiological and subjective data ensures multidimensional evidence for evaluating the effectiveness of different interaction modes, thereby providing a robust foundation for subsequent assessments.

### 3.4. Evaluation Criteria

As previously mentioned, we are investigating the impact of different interaction methods in cultural games on users’ emotional experiences. The subjective dimension uses questionnaire items to measure participants’ self-reported cultural perception and emotional involvement. Table 6 presents the questions used to assess user experience, which are categorized into three dimensions: The user experience dimension, which evaluates whether the game process is engaging under the given interaction method. The cultural understanding dimension, which measures whether the task enhances the participant’s cognition and emotional resonance with ICH. The emotional engagement dimension, which assesses whether the participant feels immersed during the task. Each question is answered using a 5-point Likert scale, with scores ranging from 1 to 5, where 1 corresponds to “very tired” and 5 corresponds to “very relaxed”.

### 3.5. Measurement

#### 3.5.1. FNIRS

**Figure 5:**
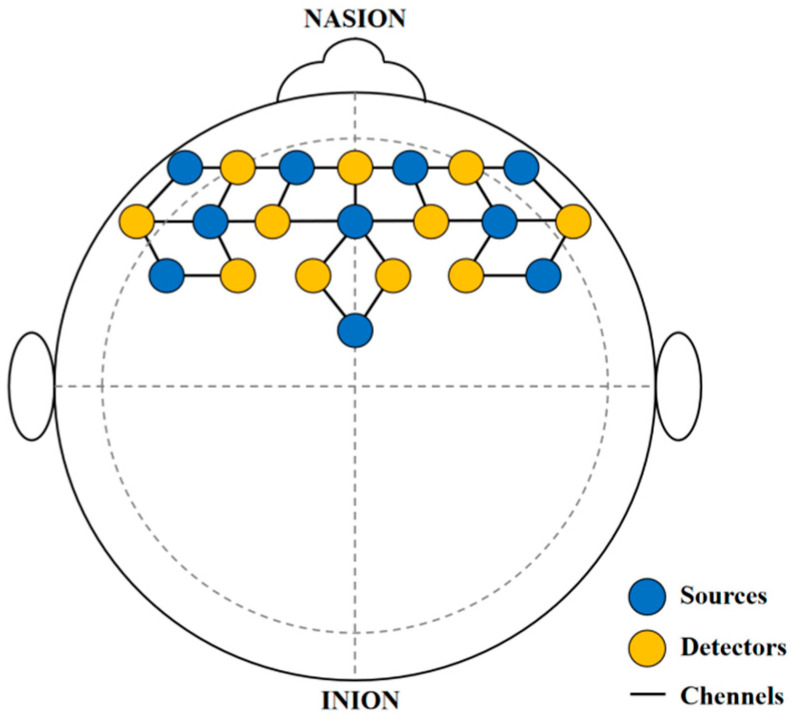
Schematic of the fNIRS cap layout design.

In this study, the brain oxygen β-values are primarily measured to reflect users’ cultural experience and their perception of ICH. For this experiment, the Photon Cap C20 system (Cortivision, Lublin, Poland) is utilized to collect real-time brain oxygen signals from participants’ prefrontal cortex regions. The sampling frequency is 10 Hz, with wavelengths of 760 nm and 850 nm. Using the 10–20 coordinate system, 21 probes (11 light sources and 10 detectors) are placed on the participants’ left and right prefrontal areas, covering three major functional regions: the orbitofrontal cortex (OFC), the ventrolateral prefrontal cortex (VLPFC), and the dorsolateral prefrontal cortex (DLPFC) [48], as shown in Figure 5. The final measure of neural activation strength is quantified by the β-value of changes in oxygenated hemoglobin concentration (HbO). Changes in HbO concentration, especially in the prefrontal cortex, are used to reflect affective responses during cultural interaction tasks, such as attention, engagement, and emotional regulation, which represent the emotional experience that culture brings to users.

The system’s accompanying software, Cortiview (version 1.11.1), is used to record the entire process, including both the interactive task segments and the corresponding resting segments. To minimize the motion artifacts, we conduct IMU (Inertial Measurement Unit) calibration through the Cortiview software. This calibration process captures real-time head motion parameters (accelerations and angular velocities across three axes) and adjusts the signal quality accordingly. The IMU module helps ensure that only stable head positions are accepted for the start of the task. During the recording, participants are also instructed to minimize unnecessary movement, especially during gesture-based tasks. Through this combination of IMU-based signal correction and behavioral control, the potential impact of motion artifacts is effectively reduced. For each interaction modality, three task repetitions are conducted, and the average value across the three trials is calculated. To control for individual baseline variability, we subtract the corresponding resting-state baseline from the averaged task-state value. This resting-state measurement serves as the emotional baseline of the participant, allowing for obtaining a baseline-normalized activation measure that more accurately reflects task-induced neural responses. This method ensures that the final values represent activation changes specifically attributable to the interaction task, rather than individual emotional or physiological differences at baseline.

#### 3.5.2. EDA

Galvanic skin response (GSR) sensors are employed in this study to monitor participants’ emotional physiological reactions. GSR signals, due to their sensitivity to the sympathetic nervous system, are widely used in research on emotional arousal, stress perception, and interactive experiences. In the experiment, ErgoLAB Human Factors Experimentation Platform and the ErgoLAB EDA Wireless Galvanic Skin Sensor (Kingfar, Beijing, China) are used to collect participants’ skin conductance signals. For the EDA indicator, the collection range is from 0 to 3 μS, with an accuracy of 0.01 μS, providing high temporal precision to ensure synchronization with events. The EDA sensors are attached to the index and middle fingers of the participant’s non-dominant hand, continuously recording the dynamic changes in skin conductance. The primary metrics record included the skin conductance (SC), tonic signal, and phasic signal, which are used to assess the intensity of emotional arousal and the activation level of the sympathetic nervous system during the gaming experience, reflecting the real-time intensity of emotional arousal during cultural interaction. After the experiment begins, EDA data are synchronously collected during both the resting and interactive phases of each task, aligned with the brain oxygen timeline. The final EDA index is obtained by subtracting the resting-state baseline from the task-state average value. This baseline-corrected EDA reflects sympathetic nervous system activation specifically induced by the interaction experience, minimizing the influence of individual emotional arousal levels at baseline.

#### 3.5.3. IRT

Infrared thermography (IRT) is used to track subtle temperature changes in facial areas, which are associated with emotional states such as stress, excitement, and engagement. The infrared camera used in our experiment is the KIR-2008z (Huajingkang Optoelectronics Technology Co., Ltd., Wuhan, China), which features a high-sensitivity, uncooled infrared focal plane detector, excellent imaging circuit components, and optical and display systems, providing superior infrared imaging performance. The camera’s optical resolution is 384 × 288 pixels, with a measurement range from 30 °C to 42 °C and a temperature measurement accuracy of ±0.3 °C. The field of view is 44.3° × 34.0°. In the experiment, participants are instructed to face the thermal camera, which is positioned 0.5 m away from them.

The application used for data collection is the KIR-2008Z Infrared Thermal Imaging Health Management System (version 5.6.0), which is capable of receiving data from the thermal camera. Based on this software, an experimental procedure to acquire thermal images is carried out. During the resting period before each game session, a thermal image is captured every 10 s, resulting in 4 thermal images per game session’s resting phase, for a total of 12 images across three game sessions. The average temperature of the region of interest (ROI) in this state is taken as the participant’s baseline temperature. After the game begins, during each of the three 50 s gameplay periods, a thermal image is captured every 10 s, resulting in 4 thermal images per 50 s period and 12 thermal images per game session. The temperature of the ROI during these periods is recorded as the experimental temperature. The final IRT measure is derived by subtracting the resting-state baseline temperature from the average task-state temperature. This baseline correction isolates the thermal responses specifically induced by the interactive task and minimizes the impact of inter-individual variability.

### 3.6. Data Preprocessing

In this study, to improve the quality of the fNIRS brain oxygen data and ensure its applicability for subsequent statistical analysis, the NIRS-KIT V3.0 Beta (a MATLAB toolbox) developed by the State Key Laboratory of Cognitive Neuroscience and Learning at Beijing Normal University is utilized for preprocessing task-related data [49]. This toolbox supports graphical operation and various standardized data processing workflows, making it suitable for near-infrared brain imaging data in brain activation research. First, raw data exported from the Cortview system is imported into MATLAB R2024a and converted into a data structure compatible with NIRS-KIT V3.0 Beta. Then, the following preprocessing steps are performed on each participant’s data: (1) The raw oxygenated HbO concentration time series is trimmed to remove irrelevant time intervals. The data is segmented based on the recorded start and end timestamps of each trial. Only the time periods corresponding to the resting phase and the task phase are retained, resulting in a total of 24 min and 30 s of usable data per participant. All unrelated intervals, such as instruction time and system initialization, are excluded to avoid introducing noise into the analysis. (2) A first-order polynomial regression model is applied to estimate the underlying linear trend in the time series. The estimated trend is then subtracted from the original HbO concentration data to eliminate slow drifts and preserve task-evoked hemodynamic fluctuations. (3) To minimize motion-related artifacts, the Temporal Derivative Distribution Repair (TDDR) algorithm is applied. This method corrects sudden spikes in the data by modeling the distribution of temporal derivatives, effectively restoring signal continuity while preserving underlying neural signals. (4) Artifacts unrelated to the experimental data are eliminated by using the filtering module in NIRS-KIT, which preserves low-frequency brain activity related to the task and suppresses high-frequency physiological noise. A 3rd-order Butterworth Infinite Impulse Response (IIR) bandpass filter is applied with a frequency range set between 0.01 Hz and 0.1 Hz. This filtering process removes high-frequency physiological artifacts such as cardiac signals and motion noise, as well as low-frequency drift. It ensures the retention of meaningful hemodynamic signals that reflect task-related neural activation, consistent with standard frequency characteristics in task-based fNIRS research [50]. After preprocessing, the data is saved in the NIRS-KIT standard format (.mat file), containing three types of hemoglobin concentration data (oxyData, dxyData, totalData), channel information, and task reference wave information. Following the preprocessing of the task-related fNIRS data, we perform individual-level statistical analysis based on the General Linear Model (GLM), extracting the β-values corresponding to task activation for each channel, which serves as an indicator of neural activation strength. The model is formulated as in (1):

(1) $$y=\beta_{1}x_{1}+\beta_{2}x_{2}+\ldots +\beta_{L}x_{L}+\varepsilon$$

In this model, y represents the dependent variable, which is the fNIRS signal from a specific observation channel. x_1_, x_2_, …, x_L_ denote the independent variables, which can be understood as the individual hemodynamic responses elicited by different task conditions. β_1_, β_2_, …, β_L_ represent the model coefficients for the independent variables, indicating the extent to which each variable contributes to the observed fNIRS signal. The portion of the fNIRS signal that cannot be explained by the explanatory variables is referred to as the residual ε. Considering all observation time points of the fNIRS signal y_1_, y_2_, …, y_T_, where T is the total number of observation points, the equation can be expressed in the following form, as in (2):

(2) $$Y=X\hat{\beta }+\varepsilon$$

In this formula, Y is the observed data matrix, X is the design matrix, $\hat{\beta }$ is the estimated parameter vector, and ε is the residual vector. Given the design matrix X and the observed data Y, the model parameters are estimated using the ordinary least squares method, as in (3):

(3) $$\hat{\beta }={\left(X^{T}X\right)}^{-1}X^{T}Y$$

X^T^ represents the transpose of X, and the parameters $\hat{\beta }$ combine the independent variables to produce predicted values of Ŷ that approximate the observed data, thereby minimizing the residual ε. The design matrix composed of all independent variables is the core of fNIRS data modeling and plays a decisive role in the quality of modeling and the accuracy of estimating individual hemodynamic response indicators [50,51]. In this study, the β_1_, β_2_, …, β_L_ derived from the model are used as the primary indicators of task-evoked neural activation. This standardized preprocessing workflow ensures the temporal consistency and comparability of the fNIRS data.

For the EDA data preprocessing, signal denoising, feature extraction, and baseline correction are carried out. Under synchronized experimental conditions, the ErgoLAB v3.17.16 records the time points for task onset, task offset, and rest states, ensuring that the physiological signals correspond accurately to the experimental conditions, which guarantees precise mapping between EDA data and task events. For the EDA data preprocessing, a standardized pipeline is implemented using the ErgoLAB Human Factors Experimentation Platform to ensure data quality and comparability. The preprocessing of raw EDA data is conducted as follows: (1) To remove high-frequency noise and enhance signal clarity, a Gaussian filter is applied for smoothing, with a window size of 5 samples. Gaussian filtering is a linear smoothing technique designed to reduce Gaussian noise in EDA signals. By modeling the signal data as an energy transformation process, where noise typically resides in the high-frequency domain, the Gaussian filter effectively suppresses noise while preserving the underlying physiological signal. Its core function is as in (4):

(4) $$G\left(x\right)=\frac{1}{\sqrt{2\pi }\sigma }e^{-\frac{x^{2}}{2\sigma^{2}}}$$

(2) The original SC signal comprises two components: a tonic component and a phasic component. The tonic component is represented by the Skin Conductance Level (SCL), which reflects the participant’s overall arousal level during a given task or resting period. For a given time interval, SCL is computed as the arithmetic mean of the SC samples in that interval, as in (5):

(5) $$SCL=\frac{1}{N}\sum_{i=1}^{N}SC_{i}$$

(3) SCR features are extracted using the SCR analysis module in ErgoLAB, with peak detection sensitivity set to medium, a maximum rise time of 4 s, a half-recovery time of 4 s, and a minimum response amplitude threshold of 0.03 μS. The SCR amplitude is computed as the peak minus baseline, as in (6):

(6) $$SCR={SC}_{peak}-{SC}_{baseline}$$

The SC_peak_ is the maximum SC value within the post-stimulus window, and the SC_baseline_ is the average SC value within the pre-stimulus baseline window. Event-related analysis windows are set to 1–4 s following the onset of each stimulus to ensure temporal relevance of SCR extraction. (4) To control for inter-individual variability, the SCL and SCR values for each task condition are baseline-corrected by subtracting the corresponding resting-state averages, resulting in ΔSCL and ΔSCR. All feature values are represented as the difference between each task round and the baseline phase to assess the relative changes induced by the interaction task. This enables cross-subject comparative analysis under different conditions, revealing how various interaction methods affect users’ physiological arousal levels.

**Figure 6:**
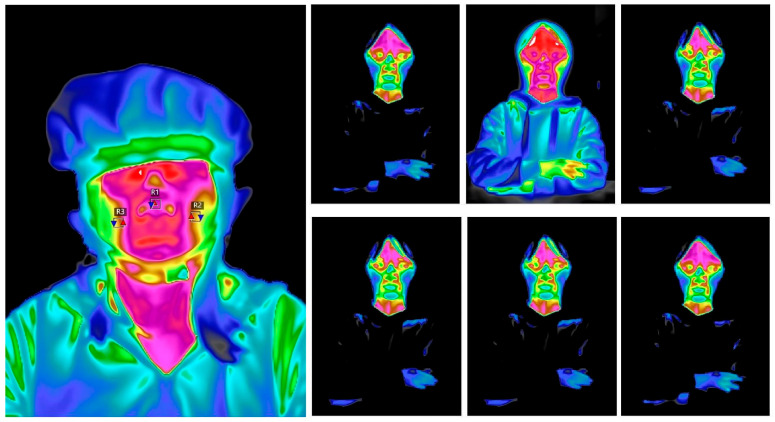
Facial ROIs for infrared thermal imaging.

For preprocessing the infrared temperature data, we first define the ROI for analysis. According to previous studies, the nasal tip and bilateral cheeks in the facial region exhibit higher physiological sensitivity and stability in emotional regulation and autonomic nervous system responses [52]. Among them, the nasal tip area, in particular, shows a significant response to sympathetic nervous system activation, with a notable decrease or increase in temperature under stress, pleasure, or alertness. Therefore, this study selects the nasal tip, left cheek, and right cheek as the primary temperature analysis areas, which have good emotional indicator validity and signal stability. A threshold of approximately 1.3 °C change in facial temperature is considered metrologically significant and consistent with prior studies in emotion thermography [53]. During the preprocessing of the temperature images, the optical detector first captures the infrared radiation signal from the target area and converts it into Analog-to-Digital (AD) data. The AD data are processed in the camera’s internal processing unit using non-uniformity correction, image filtering, sharpening, and related algorithms to transform the raw digital signal into calibrated temperature data in thermal images. In the infrared thermal images, color represents temperature, with red indicating higher temperatures and blue indicating lower temperatures. Subsequently, we use infrared thermal imaging analysis software to manually select the three ROI areas in each thermal image, with each region set as a 5 × 5 pixel window to represent the target areas, as shown in Figure 6.

Subsequently, the average temperature value of the pixels within the window is extracted to quantify the thermal change in the region. The temperature change (∆T_ROI_) is defined as a representative indicator of emotional activation level, as in (7).

(7) $$∆T_{ROI}=T_{task}-T_{baseline}$$

where T_task_ represents the average temperature of the ROI during the interaction task, and T_baseline_ is the average temperature during the resting state. The positive or negative change in ∆T_ROI_ reflects the activation level of the autonomic nervous system (particularly the sympathetic nervous system) and is used to indirectly assess the emotional arousal and psychological stress levels of the participants under different interaction conditions.

## 4. Results

### 4.1. User Perception

To compare the effects of three interaction methods (gesture, keyboard, and no interaction) on users’ subjective perceptions, we conduct a one-way ANOVA and post hoc tests on participants’ subjective ratings across three dimensions, “user satisfaction and enjoyment,” “cultural identity and understanding,” and “emotional engagement and immersion.” Before conducting the one-way ANOVA, rigorous assumption checks were carried out to ensure the validity of the analytical results. Specifically, the Shapiro–Wilk test was used to assess whether the data for each interaction mode conformed to a normal distribution across the three subjective rating dimensions. The results indicated that the data from all groups met the normality assumption (*p* > 0.05). Additionally, Levene’s test was employed to examine the homogeneity of variances, and the results confirmed that the variances across groups were homogeneous in all three dimensions (*p* > 0.05), thereby satisfying the fundamental assumptions required for ANOVA. Upon meeting these prerequisites, a one-way ANOVA was conducted with the interaction mode (gesture, keyboard, and no interaction) as the independent variable and each of the three subjective rating dimensions as dependent variables to examine the main effect of the interaction mode. The results show that the interaction method has a significant main effect across all three dimensions (*p* < 0.05, 95% CI), as shown in Table 7. Further post hoc pairwise comparisons conducted using Tukey’s HSD test demonstrated that gesture interaction significantly outperforms both keyboard interaction and no interaction across all dimensions.

The results suggest that gesture interaction, with a higher cultural fit, effectively enhances users’ emotional experience and cultural understanding, which is consistent with our core hypothesis that culturally congruent interaction methods provide greater user advantages.

### 4.2. Cortical Activation

#### 4.2.1. Cortical Activation of Channels

**Figure 7:**
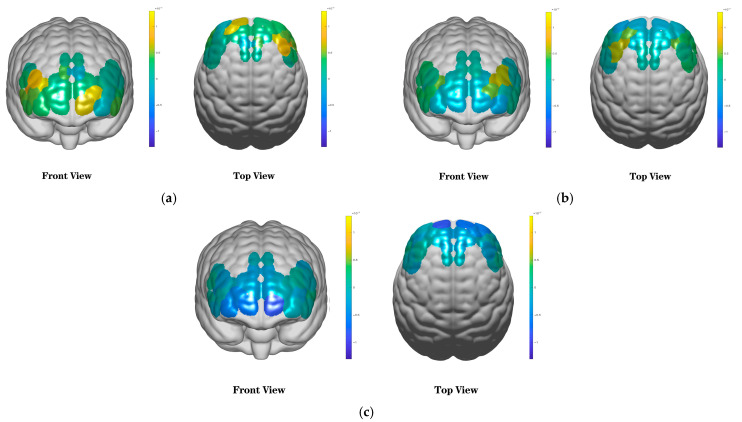
(**a**) The brain activation state in gesture interaction; (**b**) the brain activation state in keyboard interaction; (**c**) the brain activation state in no interaction.

In processing the HbO concentration data, we exclude records where high-quality fNIRS signals were difficult to obtain. Ultimately, 61 participants remained. To compare the cortical activation levels under different interaction conditions, we create heatmaps based on β-values, illustrating the activation distribution across channels for the three interaction methods. The color range of the heatmaps goes from yellow (representing positive activation) to blue (representing negative activation), with a threshold set at ±1.3 × 10^−7^, as shown in Figure 7.

From the heatmap, it is visually apparent that the cortical activation range is widest under the gesture interaction condition, with more yellow and green areas in the channel distribution, reflecting stronger activation levels. The keyboard interaction condition shows relatively weaker activation, with colors predominantly in blue and green, indicating lower brain activity involvement. In the no interaction condition, almost all channels present negative β-values, with the blue areas most concentrated in the heatmap, suggesting a low activation state in the brain. Overall, the results indicate significant differences in cortical activation levels across the different interaction methods, with gesture interaction causing the most prominent activation.

We first conducted normality tests on the HbO concentration data collected from 29 channels, specifically using the Shapiro–Wilk test. The results showed that, except for a few channels (e.g., C4, C7, C9, and C10), the data from most channels significantly deviated from the normal distribution in both tests (*p* < 0.01), indicating that the data did not meet the normality assumption. Given that parametric tests, such as analysis of variance (ANOVA), rely on strict normality assumptions, their results are prone to bias when the data fail to meet these conditions. Therefore, to ensure the validity and reliability of the statistical analysis, this study employed the Kruskal–Wallis H test, a non-parametric method. This test is applicable to scenarios involving multiple independent samples with non-normally distributed data (in this study, the interaction mode serves as the grouping variable, dividing the data into groups such as gesture interaction, keyboard interaction, and no interaction). Compared with parametric tests, the Kruskal–Wallis H test can more robustly examine the overall distribution differences among multiple independent samples when the data are non-normally distributed, avoiding result biases caused by violated distributional assumptions.

Subsequently, a non-parametric test was conducted with the three interaction modes as the grouping variable to analyze the HbO concentration data from 29 channels in the prefrontal cortex and the parietal motor cortex. The results indicated that five channels (C2, C10, C15, C18, and C24) exhibited significant differences in HbO concentration across the three interaction modes. Pairwise comparisons between groups were then performed using the Mann–Whitney U test, a non-parametric method suitable for analyzing differences between two independent samples, which was applied following a significant overall test to further identify specific between-group differences. Given that three pairwise comparisons were conducted (gesture vs. keyboard, gesture vs. no interaction, and keyboard vs. no interaction), the Bonferroni correction was applied to control the Type I error rate. Accordingly, the significance threshold was adjusted to α′ = 0.05/3 ≈ 0.0167 to ensure the rigorous determination of statistical significance.

Gesture Interaction vs. Keyboard Interaction: In channel 2 (*p* = 0.015), the activation levels for gesture interaction are significantly higher than for keyboard interaction. This suggests that, compared to traditional keyboard input, gesture interaction may better engage users’ cognitive resources and emotional experiences, especially in brain areas involved in motor control and contextual simulation.

Gesture Interaction vs. No Interaction: In channel 2 (*p* = 0.002), 10 (*p* = 0.004), 15 (*p* = 0.001), 18 (*p* = 0.006), and 24 (*p* = 0.001), the activation levels for gesture interaction are significantly higher than in the no interaction condition. This suggests that, compared to no interaction, gesture interaction triggers greater activation in the prefrontal cortex, reflecting that users may experience higher levels of cognitive load, emotional engagement, or decision-related neural activity under this interaction mode.

Further examination of the distribution of these significantly different channels in cortical space reveals that they are primarily concentrated in areas related to emotional processing, motivational evaluation, and cognitive control. This spatial distribution not only supports the advantage of gesture interaction in engaging the multidimensional neural system but also suggests that it may trigger deeper emotional and cognitive involvement in the context of cultural scenario construction. In contrast, although the heatmaps for keyboard interaction and no interaction conditions show certain visual differences, there are no significant differences in activation levels across all channels in terms of statistical significance. This indicates that the differences in cortical resource engagement between the two conditions are relatively limited and may not effectively stimulate higher-level integrative processing functions in the prefrontal cortex, which in turn affects the user’s immersion experience and active engagement.

#### 4.2.2. Cortical Activation of ROIs

**Figure 8:**
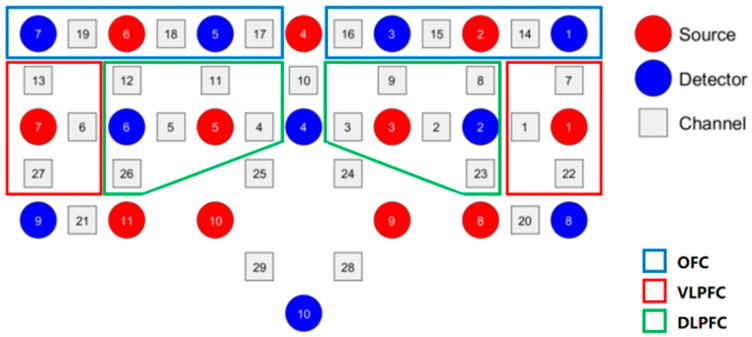
ROIs.

To further explore the differences in HbO concentration activation across larger cortical areas for different interaction methods, we construct three ROIs based on data from 29 channels, namely OFC, VLPFC, and DLPFC, as shown in Figure 8. Each ROI consists of multiple mirror–image channels from the left and right hemispheres (OFC: Channels 14–19, VLPFC: Channels 1, 6, 7, 13, 22, 27, DLPFC: Channels 2 to 5, 8 to 12, 23, 26), and the cortical activation level for each ROI is calculated by averaging the β-values across all channels within the respective ROI.

We first assessed the normality of the mean cortical activation values of three interaction modes (gesture, keyboard, and no interaction) within each region of interest (ROI) using the Shapiro–Wilk test. Since the normality assumption was not met (*p* < 0.05), we conducted non-parametric tests using the Kruskal–Wallis H test to evaluate the overall differences between different conditions. Subsequently, the Mann–Whitney U test was employed for pairwise comparisons, and the Bonferroni correction was applied to control Type I errors, with the significance threshold adjusted to α′ ≈ 0.0167. The results indicate significant differences in HbO activation levels between the different interaction modes across the three ROIs (see Table 8), as detailed below.

Gesture Interaction vs. Keyboard Interaction: In the OFC (*p* = 0.010), the activation level for gesture interaction is significantly higher than that for keyboard interaction, whereas no significant difference is found in the VLPFC or DLPFC. This suggests that gesture interaction may more effectively engage brain areas associated with higher-order decision-making and attention control when handling culturally related tasks, while keyboard interaction shows a narrower activation range.

Gesture Interaction vs. No Interaction: In the OFC (*p* = 0.006), VLPFC (*p* = 0.010), and DLPFC (*p* = 0.005), the activation levels for gesture interaction are significantly higher than those for the no interaction condition. This result indicates that gesture interaction, compared to no interaction, triggers broader activation of the prefrontal cortex, involving neural processes related to decision-making, reward anticipation, action control, and emotional evaluation. This may reflect a higher level of user engagement and emotional involvement in this mode.

Keyboard Interaction vs. No Interaction: No significant differences in activation are observed between keyboard interaction and no interaction in any of the three ROIs. This suggests that traditional input methods may not significantly enhance the user’s neural engagement in the task, particularly in regions associated with emotional or cultural cognitive processing.

Overall, gesture interaction shows the highest brain oxygen activation levels across all three key prefrontal ROIs, further supporting its potential to provide more natural and immersive interactive support in contextualized, concrete cultural experience scenarios, thereby eliciting stronger emotional involvement and cognitive processing.

#### 4.2.3. Inter-Channel Activation Covariance Analysis

To investigate the coordination patterns of cortical activation levels across different brain regions under various interaction modes, a Pearson correlation analysis was conducted on the β-values of 29 fNIRS channels for each of the three interaction modes, resulting in a total of 406 channel pairs (29 × 28/2).

**Figure 9:**
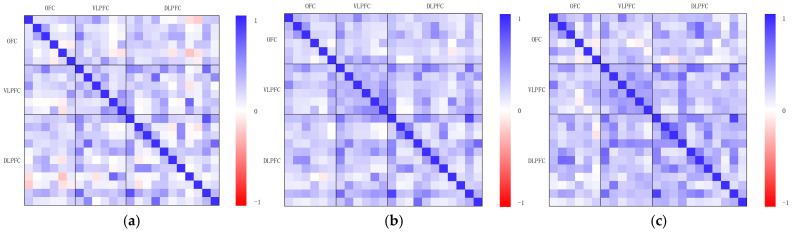
(**a**) Correlation heatmap of β-values between channels under the gesture-based interaction mode. (**b**) Correlation heatmap of β-values between channels under the keyboard interaction mode. (**c**) Correlation heatmap of β-values between channels under the non-interactive mode.

Figure 9 presents the correlation heatmaps under the three interaction modes. The heatmaps are symmetric along the diagonal from the top-left to the bottom-right. Each pixel in the 29 × 29 matrix represents the Pearson correlation coefficient between a pair of channels, with blue indicating +1, red indicating −1, and white indicating 0, along with gradual color transitions representing intermediate values. The channels are ordered based on their corresponding regions of interest (ROIs), specifically the OFC, VLPFC, and DLPFC. The three ROIs are delineated by gaps forming a 3 × 3 sub-matrix structure within the heatmap.

The comparison reveals that under the gesture interaction condition, the heatmap exhibited a relatively even distribution of color blocks, particularly within the DLPFC and between the DLPFC, OFC, and VLPFC regions. This pattern indicates widespread and flexible coordination patterns, reflecting more diverse and dynamically regulated cortical co-activation across multiple brain areas. In contrast, for the keyboard interaction condition, the darker color blocks were concentrated in specific regions, mainly within the OFC and parts of the DLPFC. This distribution represents a more stable but limited activation pattern, with moderate coordination strength, suggesting relatively restricted cognitive and emotional regulation demands. For the non-interactive condition, the darker color blocks were primarily confined within the OFC, VLPFC, and DLPFC regions, with markedly reduced cross-regional coordination. The activation pattern appeared more homogeneous and lacked dynamic variation.

### 4.3. EDA Data Analysis

**Figure 10:**
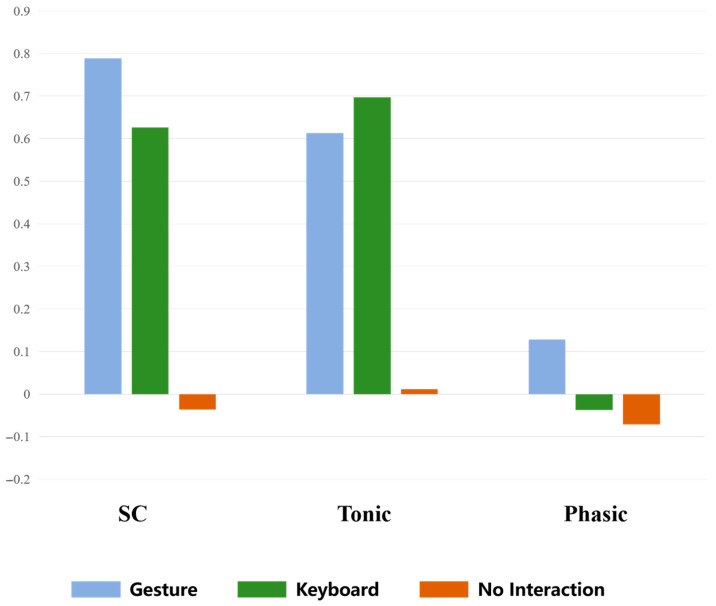
Mean values of EDA indicators under three interaction modes.

To assess the physiological arousal levels under different interaction modes, this study analyzes three key indicators in skin conductance: SC, the tonic component, and the phasic component. Data are preprocessed using the EDA signal processing tool, removing artifacts, and then extracting the mean values for the total SC, the mean value of the tonic baseline trend component, and the mean value of the phasic rapid fluctuation response component. To analyze electrodermal activity (EDA), the mean value for each interaction condition (gesture, keyboard, and no interaction) was first calculated across all participants. Since the EDA signals did not follow a normal distribution, thereby violating the assumption of normality required for parametric tests, the Kruskal–Wallis H test was employed. When a significant overall effect was observed, pairwise comparisons were conducted using the Mann–Whitney U test to identify specific differences between conditions. Additionally, the Bonferroni correction was applied to control for Type I error, adjusting the significance threshold to α′ = 0.05/3 ≈ 0.0167 accordingly. The results, as shown in Table 9, indicate significant differences across the three indicators (SC: *p* < 0.001; tonic: *p* < 0.001; phasic: *p* < 0.001) between different interaction modes. Further post hoc testing reveals that gesture interaction significantly differs from no interaction across all indicators, while keyboard interaction also significantly differs from no interaction in SC (*p* < 0.001), tonic (*p* < 0.001), and phasic (*p* = 0.005). No significant differences are found between gesture interaction and keyboard interaction. Figure 10 illustrates the distribution of mean values for the three skin conductance indicators under the three interaction modes, visually depicting the impact of different interaction modes on the skin conductance response.

### 4.4. Infrared Temperature Data

This experiment analyzes the effect of different interaction modes on facial temperature. Infrared thermography is used to investigate the physiological differences in users across various experiences. Temperature change is defined as the difference between the average temperature during rest and the average temperature during the three interaction modes. Three indicators are used as dependent variables: the temperature change at the nasal tip, the temperature change on the right cheek, and the temperature change on the left cheek. To ensure the integrity of facial temperature data, we exclude samples with missing data due to non-capture by the infrared camera and samples with anomalous temperature data from specific ROIs, leaving a total of 34 subjects. We observe that the temperatures of the three facial ROIs change when users perform tasks under the three different interaction modes. Prior to conducting the analysis of variance (ANOVA), the prerequisites for the analysis must be verified. First, the Shapiro–Wilk test was employed to assess the normality of data across the three regions of interest (ROIs) under each interaction condition. The results indicated that the data conformed to a normal distribution (*p* > 0.05), thus satisfying the basis for parametric testing. Second, Levene’s test was employed to verify the homogeneity of variances, with “based on the mean” selected as the test type. The results confirmed that the variances across all ROI dimensions were homogeneous under the three interaction conditions (*p* > 0.05), ensuring the validity of the subsequent one-way ANOVA results. A one-way ANOVA test was applied to examine group differences among the three interaction modes, with interaction mode (comprising three levels: gesture interaction, keyboard interaction, and no interaction) as the grouping independent variable, and temperature changes in the nasal tip, right cheek, and left cheek as the dependent variables, respectively. Separate models were constructed for each region of interest (ROI) for analysis. The results indicated that significant effects were observed in the nasal tip, right cheek, and left cheek, as shown in Table 10 (*p* < 0.05, 95% CI). Subsequently, Tukey’s HSD test was employed for post hoc analyses to further compare the differences between groups. The nasal tip temperature shows significant differences between gesture interaction and keyboard interaction, as well as between gesture interaction and no interaction (*p* < 0.01; *p* < 0.05). The right cheek temperature also exhibits significant differences between gesture interaction and keyboard interaction and between gesture interaction and no interaction (*p* < 0.01; *p* < 0.05). The left cheek temperature shows a significant difference between gesture interaction and no interaction (*p* < 0.05).

Temperature changes exhibit consistency across the three interaction modes, with the most significant temperature change occurring during gesture interaction, followed by keyboard interaction, and the smallest temperature change during no interaction. During finger-based interaction, the facial ROIs of users show a temperature clearly higher than the baseline temperature, while during keyboard interaction, the nasal tip and right cheek temperatures are lower than the baseline, and no significant changes are observed during no interaction, as shown in Table 11.

**Figure 11:**
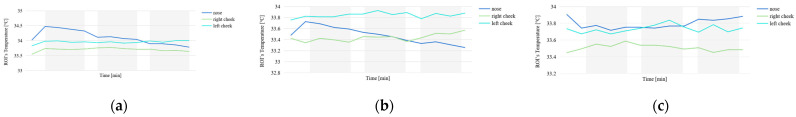
Temperature changes in facial ROIs during (**a**) gesture interaction; (**b**) keyboard interaction; (**c**) no interaction.

From Figure 11, we observe that during finger interaction, the temperature at the nasal tip rises and then falls across three stages, with a gradual decrease in temperature throughout the interaction. Temperature changes in the cheeks are minimal. During keyboard interaction, the nasal tip temperature shows an overall decreasing trend, but with a smaller change compared to gesture interaction. When users watch a video, there are no significant temperature changes in the three regions of interest, with nasal tip, right cheek, and left cheek temperatures remaining at 33.8 °C, 33.5 °C, and 33.7 °C, respectively.

## 5. Discussion

### 5.1. Major Findings

#### 5.1.1. User Perception

Gesture-based interaction demonstrates significantly higher performance in subjective evaluation metrics such as user satisfaction, cultural identity, and emotional immersion compared to keyboard-based interaction and non-interactive modes. This advantage stems from its inherent alignment with the traditional manipulation techniques of Chinese marionette puppetry. Unlike conventional input methods such as keystrokes or passive observation, gesture interaction more closely mirrors the expressive mode of “conveying emotion through hand movements” and “expressing meaning through form,” which are fundamental to this intangible cultural heritage. This alignment between physical actions and cultural context fosters stronger cultural resonance and immersion during user interaction, thereby enhancing the overall quality of the subjective experience.

Moreover, this study further validates the critical role of cultural consistency in interaction design. We observe that even when interaction modes serve similar functional purposes (e.g., keyboard vs. gesture), differences in cultural expression can significantly affect user evaluations. This finding suggests that cultural elements are not merely esthetic embellishments at the content level but serve as pivotal factors influencing interaction acceptance and user perception. This is particularly important in systems designed for specific cultural communities or heritage preservation purposes.

#### 5.1.2. Cortical Activation

1. Cortical Activation of Channels

In terms of channel-level activation, the gesture-based interaction mode elicits significantly higher activation across multiple prefrontal cortex (PFC) channels compared to keyboard interaction and the non-interactive condition. Notably, the differences are most prominent in channels 2, 10, 15, 18, and 24.

The prefrontal cortex, particularly the dorsolateral and ventrolateral regions, is widely associated with higher-order cognitive functions, goal-directed behavior, and interactive experiences within social contexts [54]. The observed differences in activation across channels suggest that gesture-based interaction may drive users to engage with more cognitive resources, involving deeper motor simulation and contextual processing. This leads to heightened cortical responses at the neural level.

In the context of the marionette-themed digital game, gesture interaction enhances immersion and bodily engagement by simulating authentic performance gestures. Such embodied interaction appears to facilitate richer internal simulation and more effective allocation of attentional resources. In contrast, no significant activation differences are observed between the keyboard-based and non-interactive conditions, indicating that traditional interaction methods with merely formal input may be limited in eliciting engagement of emotional and cognitive systems in the brain.

2. Cortical Activation of ROIs

At the level of regions of interest (ROIs), gesture-based interaction demonstrates significant neural activation advantages. Compared with keyboard and non-interactive modes, gesture interaction elicited stronger activation in key prefrontal regions, including the OFC, DLPFC, and VLPFC. These ROI-level findings corroborate the channel-wise results discussed earlier, further indicating that gesture interaction more broadly recruits brain regions associated with emotional and cognitive processing.

Specifically, the significant activation of the OFC suggests that gesture interaction engages processes related to emotion regulation, value evaluation, and contextual perception. Given the OFC’s close association with positive emotional experiences, its heightened activity implies that users may achieve a higher level of emotional engagement during embodied and culturally grounded interactions [55]. This neural pattern supports the subjective evaluation results showing that gesture interaction delivers a superior emotional experience.

The DLPFC, known as a central hub for cognitive control and task planning, also shows stronger activation under gesture-based interaction. This indicates that users engage in higher-order cognitive functions such as goal management, working memory retrieval, and action planning. These cognitive demands often co-occur with stronger immersion and emotional involvement, aligning with the cultural resonance and emotional efficacy perceived in the gesture interaction condition.

Regarding the VLPFC, the observed activation differences between gesture-based and non-interactive conditions further demonstrate that embodied interaction triggers neural responses associated with attention control and inhibitory processing. Although the difference between gesture and keyboard interaction in the VLPFC was not statistically significant, the trend still favors gesture interaction, suggesting potential advantages in contextual adaptation and motivational engagement.

In contrast, no significant differences are observed between the keyboard and non-interactive conditions across any ROI. This neural “silence” reinforces the limitations of traditional interaction methods in evoking cultural immersion and emotional arousal—lacking both action semantics and bodily embedding, such interactions fail to activate deeper neural processes.

Overall, the ROI results provide a clear neural basis for the observed subjective advantages of gesture interaction: the broader and deeper activation of prefrontal regions reveals a compound effect on emotional resonance, cognitive resource allocation, and attentional focus. This neural mechanism underpins the enhanced affective experience during culturally themed tasks and underscores the critical importance of culturally congruent interaction design.

3. Inter-Channel Activation Covariance Analysis

The inter-channel β-value correlation analysis reveals distinct co-activation patterns within the prefrontal cortex under different interaction modalities, offering a novel perspective on how brain regions functionally integrate in response to task demands [56]. Although this analysis does not capture time-resolved functional connectivity in the traditional sense, it reflects structural covariance across regions, shedding light on how cognitive resources are mobilized and integrated under various interaction conditions.

Among the three interaction modes, gesture-based interaction elicited the uniform inter-channel covariance, indicating a higher level of functional coordination across prefrontal regions. This suggests that, compared to keyboard input or passive observation, gesture interaction engages broader neural networks and facilitates cross-regional integration in the prefrontal cortex. Such widespread synchronization implies greater cognitive resource demands, likely due to the embodied, expressive, and contextually meaningful nature of gesture-based tasks.

In contrast, the keyboard interaction condition primarily exhibits intra-regional correlations centered within the OFC, potentially reflecting localized processing related to motivation, affective evaluation, or simple decision-making. The non-interactive condition, meanwhile, shows sparse inter-channel correlations with weaker cross-regional connectivity, indicating lower overall engagement and minimal functional integration.

These results underscore the significant influence of interaction modality on the functional architecture of the prefrontal cortex. The findings support a positive relationship between the complexity of interaction and the extent of cortical resource integration—suggesting that culturally meaningful and physically embodied interaction modes such as gestures not only enhance user experience but also promote more extensive and coherent neural processing patterns.

#### 5.1.3. EDA

In terms of overall trends, both skin conductance (SC) and phasic components are significantly higher under the gesture-based interaction condition compared to the non-interactive condition. This suggests that gesture interaction elicits a stronger physiological arousal response during task execution. Conversely, SC and tonic values remain lowest in the non-interactive condition, indicating that participants maintain a relatively calm and passive physiological state when not engaged in interaction.

Non-parametric tests of SC values reveal highly significant differences (*p* < 0.001), with both gesture vs. non-interaction and keyboard vs. non-interaction conditions showing significant contrasts. However, no statistical difference is observed between gesture and keyboard interactions. This indicates that the presence of interaction itself is sufficient to elevate physiological arousal, while the specific modality (gesture vs. keyboard) plays a less critical role in modulating SC.

Regarding the tonic component, the keyboard interaction condition exhibits the highest mean tonic values, followed by gesture interaction, with the non-interactive condition being the lowest. This pattern may reflect the sustained autonomic nervous system activation required for keyboard-based tasks. Although the difference between gesture and keyboard interactions is not statistically significant (*p* = 0.718), both gesture vs. non-interaction and keyboard vs. non-interaction comparisons showed significant differences (*p* < 0.001), reinforcing the notion that interaction engages baseline autonomic regulation more effectively than passive observation.

Phasic activity further supports these findings, with significantly higher values observed in the gesture interaction condition compared to the non-interactive condition (*p* < 0.001). This suggests that gesture-based interaction is more likely to trigger rapid, short-term physiological responses. The difference between keyboard and non-interaction conditions also reached significance (*p* = 0.005), implying that phasic components are more sensitive to immersive and dynamic interaction forms such as gestures and keyboard.

#### 5.1.4. Thermal Imaging

Based on the results obtained from infrared data, it has been observed that each participant responds differently to the same game task. However, a similar trend of facial thermal responses is evident among the majority of participants. Significant differences in facial temperature changes have been found across three ROIs when comparing gesture-based interaction with non-interactive tasks. Furthermore, compared with keyboard interaction, gesture-based interaction shows significant temperature differences in the tip of the nose and the right cheek areas. Significant differences in facial temperature changes have been observed across three ROIs when comparing gesture-based interaction with non-interactive conditions. When comparing gesture-based interaction with keyboard-based interaction, significant thermal differences appear in the tip of the nose and the right cheek areas. During gesture interaction, temperature increases are evident in all three ROIs, with the most pronounced rise occurring in the nasal tip region [57].

Thermal changes in the nasal area reflect the vasoconstriction and vasodilation controlled by central nervous system (CNS) activation mediated through the sympathetic nervous system (SNS). Compared to the cheeks, the nasal region has a thinner dermal layer, making it more sensitive to changes in blood flow. Therefore, when game tasks stimulate the autonomic nervous system (ANS), the acceleration of facial blood circulation leads to more notable temperature changes in the nasal region. The relatively larger thermal variations induced by gesture interaction, compared to keyboard interaction or passive video viewing, may be attributed to factors such as emotional responses and physical engagement.

Trend analyses of average temperature changes reveal variations in users’ emotional valence and arousal levels under different interaction modes. The temperature of the nasal tip exhibits a rise-then-fall pattern across all phases of gesture-based and keyboard-based interaction, with a greater decline observed during gesture interaction. According to the Arousal Effect Theory, facial thermal responses correlate more strongly with arousal levels than emotional valence. The more arousing the stimulus, the more intense and rapid the thermal response [58,59]. Hence, gesture interaction appears to elicit higher arousal levels in users. Its complex and large-scale movements may quickly provoke emotional fluctuations and promote cognitive engagement, leading to increased SNS activity and a temporary rise in temperature.

By contrast, keyboard interaction, due to its simplicity and familiarity, results in lower levels of arousal. In the later stages of each game task, participants gradually become accustomed to the interaction method, leading to psychological relaxation and physical ease. As a result, SNS activity decreases, nasal temperature begins to drop, and physiological responses stabilize.

Under non-interactive conditions, compared to gesture interaction, users experience lower levels of emotional arousal and valence. Consequently, no substantial temperature changes are observed in the three ROIs.

When designing interaction systems based on cultural elements, the choice of interaction modality plays a critical role in shaping user experience. Gesture-based interaction, aligned with the cultural characteristics of Quanzhou Puppetry, can quickly activate emotional engagement and cognitive processing, thereby offering a highly immersive gaming experience. However, it is also necessary to manage the challenges posed by such specialized interaction forms, as high-load operations or repetitive tasks may lead to user fatigue or stress. A well-balanced design should thus ensure both immersion and usability.

### 5.2. Design Implications

Our findings provide practical guidance for designing culturally resonant and inclusive interactive systems. Specifically, we address the following: (1) enhancing cultural relevance through congruent interaction modalities (e.g., tradition-inspired gestures); (2) indications for designers; (3) implications for inclusive design, particularly for users with sensory/motor differences; and (4) extending this approach to other cultural formats (music, crafts, storytelling). We detail these cross-cutting opportunities below.

#### 5.2.1. For Interaction Design

It could be stated that the design of cultural digital interactions should be fundamentally driven by cultural characteristics, with the goal of constructing interaction models that align with users’ cultural cognition. This approach enhances users’ emotional engagement and cultural immersion. Designers are encouraged to embed culturally representative elements—such as ritualistic behaviors, linguistic conventions, visual symbols, and narrative structures—into the interaction logic, allowing users to develop a sense of cultural belonging and identity through active participation. Additionally, it is recommended to implement adaptive interaction feedback mechanisms that dynamically guide users toward emotional connections with cultural content, thereby enriching both the depth and warmth of the overall cultural experience. Furthermore, special attention should be given to users’ diverse cultural backgrounds and cultural sensitivities. Designers should seek a balanced strategy between interaction personalization and cultural universality to support broader cultural understanding and emotional resonance across different user groups.

#### 5.2.2. For Designers

Designers of cultural interactive systems could also benefit from this study. Our study offers a novel perspective for interaction designers, particularly those working in cultural domains. Unlike conventional common concerns in interaction design that tend to be technology-driven or rely on sporadic creative insights, we point out a serious consideration that is focused on the intrinsic correlation between the interaction modalities and culturally inherent characteristics. This focus is empirically supported by experimental data, providing designers with actionable insights. Specifically, we demonstrate that employing culturally congruent input modalities—such as gesture interactions that align with traditional practices—can significantly enhance emotional engagement and cultural resonance. This enables designers to develop user experiences that are both intuitively accessible and rich in symbolic meaning.

#### 5.2.3. For Inclusive Design

Inclusive design in cultural interaction systems should address the diverse physical and sensory abilities of potential users while preserving the depth of cultural immersion. The key insight is that cultural authenticity can be preserved while lowering the participation threshold. For instance, in the cultural experience of gesture interaction, multisensory feedback—such as synchronized visual cues or subtle haptic signals—can compensate for reduced perceptual channels, enhancing situational awareness and emotional engagement. Additionally, gradual, adaptive guidance can further help users acclimate to culturally specific gestures without losing the symbolic resonance of the interaction. These strategies demonstrate how inclusive design can broaden access while retaining the immersive and emotionally rich qualities of culturally rooted interfaces.

#### 5.2.4. For Other Cultural Formats

Although this study focuses on a specific form of cultural heritage interaction, the methodological approach and design principles identified here are transferable to other cultural domains, such as traditional music, handicrafts, and oral storytelling. In music-based cultural systems, interaction modalities could mimic traditional instrument-handling postures, enabling users to engage not only with the auditory content but also with the embodied cultural practices. For crafts, multimodal sensing can capture users’ fine-motor engagement, giving the user an immersive sense of operation. In oral storytelling contexts, combining speech-based interfaces with visual symbolism can replicate the rhythm, emphasis, and performative qualities of live narration. Across these varied formats, the use of culturally congruent interaction methods and symbolic alignment between interface and heritage elements can collectively foster emotional resonance, cultural respect, and experiential depth.

### 5.3. Limitations

This study has several limitations. First, this study focuses on a clearly defined participant group, young university students because in this intelligent era, young people occupy a major proportion of the audience for digital heritage experiences rather than the traditional way. It is quite important to find a truly useful way for them to access culture. This group naturally possesses higher familiarity with digital interaction modalities compared to other groups. Therefore, the findings and interpretations presented here are most relevant to this demographic. While this targeted sampling ensures internal consistency, it also means that the results may not directly extend to other populations with different age profiles, cultural backgrounds, or levels of digital literacy. Another limitation lies in the relatively narrow experimental setting, which focused on a single interaction mode within a specific cultural context. This restricts the ability to compare how combinations of diverse cultural elements and interaction types affect user experience. Future research could explore the emotional and cultural impacts of digital heritage design across multicultural contexts and through wider interaction modalities, such as haptic, voice-based, or immersive visual interfaces.

## 6. Conclusions

This study explores the impact of culturally congruent interaction design on young university users’ emotional and cultural experiences by integrating cultural characteristics with interaction design. The experimental results show that digital designs with more distinct cultural features and interaction methods that align with users’ existing cultural cognition are more likely to evoke positive emotional responses and a sense of cultural identity. This finding provides empirical support for interaction design in a cultural activation context, indicating that interaction methods should not detach from their cultural context, but should fully consider users’ cultural psychological foundations and emotional resonance mechanisms.

Furthermore, this study also finds that cultural factors in relevant designs do not merely remain on the content level but act as important variables influencing users’ perceptions and behaviors. In culturally immersive interaction design, the degree to which cultural elements (such as language, action logic, and visual symbols) integrate with interaction mechanisms significantly affects users’ acceptance of the system and their deeper cultural understanding. This conclusion provides theoretical references and practical insights for fields such as digital cultural heritage preservation, cultural heritage activation design, and immersive cultural tourism experience systems.

In future work, we aim to expand the diversity of user samples beyond the university student population, introducing a broader range of cultural backgrounds and age groups to verify the acceptance levels and differential responses to cultural interaction methods among different populations. We will also enrich the dimensions and complexity of interaction methods, combining multimodal interactions (such as motion capture and haptic feedback) and multisensory stimuli to explore richer cultural experience pathways.

## Figures and Tables

**Table 1:** Demographic profiles of participants.

Category	Question	Result
Demographics	Gender	Male (25), Female (36)
	Age	Under 18 (5), 18–25 (52), Over 25 (4)
	Education Degree	Undergraduate (25), Master (33), Doctor (3)
	Major	Industrial Design (19); Art (7); Electronic Information Technology (5); Materials and Chemicals (5); Other Majors (25)
Cultural Knowledge	Cultural Familiarity	M = 2.18; SD = 0.74
	Cultural Interest	M = 3.29; SD = 0.76
Gaming Experience	Weekly Gaming Time	M = 11.37 h; SD = 10.46
	PC/Console Game Experience	Yes (75.41%), No (24.59%)
	Interactive Device Type Usage	Mouse (96.72%), Keyboard (96.72%), Joystick (68.85%), Motion Sensor (18.03%), VR (29.51%)

**Table 2:** Overview of experts.

Expert	Institute	Role	Years of Expert Experience
Expert A	Quanzhou String Puppet Troupe	Former troupe leader and performer	45
Expert B	Beijing Puppet Troupe	Veteran performer	18
Expert C	China Artists Association	Cultural Heritage Research Specialist	42

**Table 3:** The structure of the interviews.

Themes	Questions
Background Information	What is your present role or status related to Puppetry culture?
	How many years have you been involved in puppet cultural heritage?
Puppetry Gestures	What do you think about the alignment of gestures with traditional cultural characteristics?
Overall Narrative	What do you think about the faithfulness of the narrative expression?
Digital Form	What do you think about the appropriateness of redesigning puppetry for digital contexts in this form?

**Table 4:** Usability evaluation scale.

Stage	Dimension	Item/Scale	Cronbach’s α
Pre-use	Learnability	L1: The design of this interaction made it easy for me to understand. (positive)	0.746
		L2: The operation of this interaction was simple, straightforward, and easy to grasp. (positive)	
		L3: My understanding of this interaction improved over time. (positive)	
During use	Cognitive Load	C1: Mental demand–How much mental activity (e.g., thinking, deciding) was required to accomplish the task?	0.750
		C2: Physical demand–How much physical effort was required to accomplish the task?	
		C3: Temporal demand–How much time pressure did you feel while accomplishing the task?	
		C4: Performance–How successful do you think you were in accomplishing the task? (positive)	
		C5: Effort–How hard did you have to work to accomplish the task?	
		C6: Frustration–How insecure, discouraged, irritated, or stressed did you feel during the task?	
Post-use	Fatigue	F1: Overall fatigue–The overall level of fatigue you experienced.	0.814
		F2: Physical fatigue–The level of physical tiredness you experienced.	
		F3: Mental fatigue–The level of mental tiredness you experienced.	
		F4: Energy level–The extent to which you felt a lack of energy.	
		F5: Motivation loss–The extent to which you felt a decrease in motivation for daily activities.	

**Table 5:** Descriptive statistics and test results.

Dimension	Item	*p*	Z
Learnability	L1 *	0.000	−4.992
	L2 *	<0.001	−3.370
	L3 *	0.000	−5.649
Cognitive Load	C1	0.919	−0.135
	C2 *	0.012	−2.557
	C3	0.297	−1.032
	C4 *	0.000	−4.717
	C5 *	<0.001	−3.364
	C6 *	<0.001	−3.521
Fatigue	F1 *	0.000	−4.543
	F2 *	0.000	−4.613
	F3 *	0.000	−4.321
	F4 *	0.000	−6.511
	F5 *	0.000	−7.228

* The significant difference that is also supported by the medium or large effect size is in boldface and marked with *.

**Table 6:** User experience subjective questionnaire.

Evaluation Dimension	Satisfaction Interest Closeness Indicator	Description
User satisfaction and the fun of the experience	Satisfaction	I am satisfied with this interactive puppet digital game
	Interest	I think this interactive experience is very interesting
	Closeness	The interactive design makes it easy to understand and full of storytelling
	Novelty	This interactive method makes me feel novel and unique, as if I were in a real puppet scene
	Personalization	I can personalize this interaction to suit my personal preferences
	Fulfillment	Through this interactive method, I learned new intangible cultural heritage knowledge and felt the rich cultural atmosphere
	Exploration	This interactive method demonstrates the intangible cultural heritage skills through gameplay, which makes me want to explore
Cultural identity and cultural understanding	Sense of scene	This interactive method allows me to experience the rich puppet scene
	Identification	I feel more connected to puppet culture when using this interaction
	Sense of value	I think this interactive method makes me feel the value of puppet culture
	Intangible cultural heritage content	Through this interactive method, I learned about the rich and in-depth culture of puppets
	Intangible cultural heritage	This interactive method combines modern technology with traditional puppet culture
	Integration sense of involvement	This interactive method gives me the feeling of actually operating a puppet
	Cultural fit	This interactive method is very consistent with the cultural characteristics of puppets
	Cultural understanding	This interactive method has improved my understanding of puppet culture
Emotional involvement and immersion level	Emotional resonance	I was able to deeply feel the cultural emotions behind the puppets when experiencing this interactive method
	Memorability	After the interactive experience ends, I can still retain the memory of the experience
	Immersion	When experiencing this interactive method, I felt completely immersed in the world of the puppets
	Flow experience	The interactive method is very attractive, and the game level is well-designed, making people linger
	Storytelling	This interactive method allows me to immerse myself in the story scenes of the puppet show
	Learnability	The interactive mode is simple, straightforward, and easy to understand
	Usefulness	This interaction method can improve my cultural literacy

**Table 7:** Comparison of user’s subjective feelings.

Evaluation Dimensions	Significant Pairings	Difference in the Average Values	Significance
User satisfaction and experience enjoyment	Gesture vs. Keyboard	4.723 *	<0.001
	Gesture vs. No Interaction	9.390 *	<0.001
	Keyboard vs. No Interaction	4.667 *	<0.001
Cultural identity and cultural understanding	Gesture vs. Keyboard	7.897 *	<0.001
	Gesture vs. No Interaction	13.605 *	<0.001
	Keyboard vs. No Interaction	5.708 *	<0.001
Emotional engagement and immersion	Gesture vs. Keyboard	3.675 *	0.05
	Gesture vs. No Interaction	8.698 *	<0.001
	Keyboard vs. No Interaction	5.023 *	<0.001

* The significant difference that is also supported by the medium or large effect size is marked with *.

**Table 8:** Comparison of changes in β-values.

ROI		OFC	VLPFC	DLPFC
Gesture Interaction (×10^−8^)		3.391	3.819	4.736
Keyboard Interaction (×10^−8^)		−0.610	2.387	2.317
No Interaction (×10^−8^)		2.912	0.633	0.804
Gesture Interaction vs. Keyboard Interaction	Difference (×10^−8^)	4.001	1.433	2.419
	z	−2.594	−1.887	−2.056
	p	**0.0** 10 *	0.059	0.040
Gesture Interaction vs. No Interaction	Difference (×10^−8^)	6.303	3.187	5.540
Keyboard Interaction vs. No	z	−2.755	−2.576	−2.786
	p	**0.00** 6 *	**0.010** *	**0.005** *
Keyboard Interaction vs. No Interaction	Difference (×10^−8^)	2.302	1.754	3.120
	z	−0.253	−0.530	−0.463
	p	0.800	0.596	0.643

* The significant difference that is also supported by the medium or large effect size is in boldface and marked with *.

**Table 9:** Non-parametric test results of skin electrical indicators under three interaction modes.

Indicators	Significant Pairings	Significance
SC	Gesture vs. Keyboard	0.329
	Gesture vs. No Interaction	<0.001
	Keyboard vs. No Interaction	<0.001
Tonic	Gesture vs. Keyboard	0.718
	Gesture vs. No Interaction	<0.001
	Keyboard vs. No Interaction	<0.001
Phasic	Gesture vs. Keyboard	0.073
	Gesture vs. No Interaction	<0.001
	Keyboard vs. No Interaction	0.005

**Table 10:** Post hoc test.

ROIs	F	Significance
Nose	9.96	0.000 *
Right cheek	3.43	0.036 *
Left cheek	7.54	0.001 *

* The significant difference that is also supported by the medium or large effect size is in boldface and marked with *.

**Table 11:** Mean value of ROI temperature differences.

ROIs	Gesture Interaction	Keyboard Interaction	No Interaction
Mean value of nose temperature difference/°C	0.42	−0.23	0.11
Mean value of right cheek temperature difference/°C	0.19	−0.11	−0.04
Mean value of left cheek temperature difference/°C	0.22	0.08	−0.01

## Data Availability

Data are contained within the article.
